# Beyond the guidelines: participants’ perspectives on sustained MPDSR implementation in Nigeria, North Macedonia, and Sri Lanka

**DOI:** 10.1186/s12884-025-08180-0

**Published:** 2025-10-06

**Authors:** Francesca Palestra, Claudia Hanson, Eleonora Mognato, Shittu Oladapo, Kapila Jayaratne, Gligor Tofoski, Merlin Willcox, Pius Okong, Allisyn Moran, Matthews Mathai, Pamela Mazzocato, Anna Bergstrom

**Affiliations:** 1 Department of Global Public Health (GPH), Karolinska Institutet, Stockholm, Sweden; 2 Médecins Sans Frontières, Rome, Italy; 3 College of Medicine, Federal University of Health Sciences, Otukpo, Nigeria; 4 Department of Community Medicine, Faculty of Medicine, University of Colombo, Colombo, Sri Lanka; 5 University Clinic of Gynecology and Obstetrics, University St. Cyril and Methodius, Skopje, North Macedonia; 6 Primary Care Research Centre, University of Southampton, Southampton, UK; 7 MK Postgraduate School, Uganda Martyrs University, Kampala, Uganda; 8 Department of Maternal, Newborn, Child, Adolescent Health and Aging, World Health Organization, Geneva, Switzerland; 9 Consultant in Global Maternal and Perinatal Health, St John’s, NF Canada; 10 Department of Learning, Informatics, Management and Ethics (LIME) Karolinska Institutet, Stockholm, Sweden; 11 Department of Women’s and Children’s Health, Uppsala University, Uppsala, Sweden

**Keywords:** Maternal and perinatal death surveillance and response, Death review, Maternal health, Newborn health, Quality improvement

## Abstract

**Background:**

Maternal and perinatal death surveillance and response (MPDSR) was developed as a quality improvement intervention to reduce preventable maternal and newborn deaths and stillbirths. To gain deeper insight into the key components enabling sustained MPDSR implementation, we examined how MPDSR systems are organized and function in Nigeria, North Macedonia, and Sri Lanka.

**Methods:**

We conducted 61 interviews with participants who were knowledgeable about the MPDSR system of their country, including policymakers, healthcare providers, and public health officials, at the national, subnational and facility levels. We applied content analysis to inductively identify themes and categories.

**Results:**

Our findings suggest that participants perceive the goal of MPDSR as going beyond local quality improvement to encompass broader healthcare system strengthening. Four enabling components supporting sustained implementation were identified in all three countries: 1. coordination of the MPDSR “programme” through committees across levels; 2. adoption and integration of a data management and analysis system; 3. a confidential, nonpunitive approach supported by committed leadership; and 4. a multilevel, country-specific response strategy integrated with a broader health system strengthening. Sri Lanka demonstrated a highly centralized and structured approach, whereas Nigeria’s federal system showcased more diverse, multilevel stakeholder engagement. North Macedonia’s facility-based approach focused on the immediate implementation of quality improvements.

**Conclusions:**

The findings reveal that a structured, multilevel approach that is interconnected with the broader health system is supporting sustained MPDSR implementation. The potential of MPDSR as a health system programme that goes beyond facility-level mortality reduction links to an integrated health system strengthening and accountability at multiple levels.

**Supplementary Information:**

The online version contains supplementary material available at 10.1186/s12884-025-08180-0.

## Key findings

### What was known?

#### Importance of this specific problem

Maternal and Perinatal Death Surveillance and Response (MPDSR) systems are crucial for reducing preventable maternal and newborn deaths, which remain high in low- and middle-income countries (LMICs). MPDSR programs aim to go beyond simple death audits, creating an integrated approach that enhances healthcare quality and accountability, fosters learning, and supports health system strengthening. Countries have implemented MPDSR to identify contributing factors to maternal and perinatal deaths, helping healthcare systems implement necessary quality improvements. However, challenges persist in sustaining these systems, coordinating across health system levels, and securing stakeholders’ accountability.

#### Key gap to address/aim of this paper

Existing literature lacks detailed, multilevel analyses of the operationalization and sustainability of MPDSR across various healthcare settings. This study aimed to identify enabling components that contribute to sustained MPDSR implementation across different health system structures and contexts, using Nigeria, North Macedonia, and Sri Lanka as case studies.

### What was done

#### High-level method

This study utilized qualitative methods, conducting 61 in-depth interviews with key participants, policymakers, healthcare providers, and public health officials, across national, subnational, and facility levels in Nigeria, North Macedonia, and Sri Lanka. Content analysis was used inductively to identify common themes and patterns in MPDSR implementation and its integration within broader health system frameworks.

#### Novel approach or analyses

The study took a multilevel, cross-country approach to analyze the sustainability of MPDSR across diverse healthcare settings, providing new insights into enabling components of sustained MPDSR implementation. This analysis delves into participants’ perspectives within and across countries identifying enabling components of sustained MPDSR implementation and their interconnectedness.

### What was found

#### Key result finding 1

Four enabling components were identified as essential for sustained MPDSR implementation: 1. Coordination of the MPDSR “programme” through committees by and across levels; 2. Adoption and integration of a data management and analysis system; 3. A confidential, nonpunitive approach supported by committed leadership; 4. A multilevel, country-specific response strategy integrating to broader health system strengthening.

#### Key result finding 2

Participants’ engagement and MPDSR approaches differed based on healthcare system structures. For example, Sri Lanka exhibited a highly centralized and structured approach, Nigeria demonstrated multilevel engagement within its federal system, and North Macedonia’s facility-level focus supported immediate quality improvements. Effective MPDSR requires robust communication, strong leadership, and harmonized actions across healthcare levels.

### What are the implications?

#### Action in programmes and/or measurement now

The findings suggest that successful sustained MPDSR implementation requires formal institutionalization through national policies and guidance on how to support operationalization at national and subnational levels, beyond facility level. Developing multilevel guidelines can enhance MPDSR’s integration with existing Quality of Care (QoC) and health system strengthening programs, enabling consistent quality improvements across healthcare levels and settings.

#### Future research priorities

Future research should focus on evaluating long-term impacts of MPDSR on maternal and perinatal health outcomes, understanding team dynamics within MPDSR committees, and identifying best practices for stakeholder engagement and leadership. Additionally, investigating sustainable funding models and frameworks for MPDSR integration into QoC initiatives would support broader scalability and system alignment in LMICs.

## Background

Maternal and perinatal mortality represent persistent global health challenges despite significant advances in healthcare technologies and practices. Globally, in 2023, approximately 700 women lose their lives daily due to preventable complications related to pregnancy and childbirth [1]. In addition, 2.3 million newborn deaths and 2 million stillbirths occur every year, with more than 40% of all stillbirths attributable to complications of labor [2, 3]. It is estimated that approximately half of maternal deaths and 58% of newborn deaths could be prevented with improved quality of care [4].

### Introduction to Maternal and Perinatal Death Surveillance and Response (MPDSR)

Maternal and perinatal death surveillance and response (MPDSR) is a surveillance and quality-of-care system designed to improve health outcomes for women and newborns [5, 6]. Studying adverse events as a quality improvement approach has been established in medical care for centuries. Since early 2000, maternal death reviews have been promoted in low- and middle-income countries (LMICs) by the World Health Organization (WHO) [7]. Since its inception until its transition to MPDSR, the system has been described as a system at multiple levels, from the national down to the community level [8]. The MPDSR system aims to systematically review fatalities through six steps, which include identifying, notifying, reviewing, and analyzing deaths, followed by recommending and implementing changes to address preventable factors. By examining the causes and circumstances of maternal and perinatal deaths, MPDSR seeks to identify gaps in service delivery and prevent future fatalities through the implementation of a response plan [8]. MPDSR should align with a broader quality improvement strategy, as in the case of Ethiopia, where recommendations and solutions arising from the MPDSR system have been tested and implemented through team-based quality improvement (QI) cycles using, for example, the Plan‒Do‒Study‒Act (PDSA). In some states in Nigeria, quality of care (QoC) teams and MPDSR committees have been combined, harmonizing processes and monitoring and reporting mechanisms [5].

The MPDSR system has been adapted and refined to include neonatal deaths and stillbirths in the last decade [9–11] and is currently nationally adopted in 79% of WHO member countries [12]. The primary focus of the latest WHO MPDSR operational guidance [13] is to support the establishment of MPDSR systems at the facility level.

### Progress in MPDSR implementation in the last decade

MPDSR has shown potential in reducing maternal and newborn deaths, with studies indicating reductions of about one-third [14, 15]. However, implementation faces barriers such as incomplete documentation, weak leadership, blame culture, and staff shortages [16–20]. As a complex system aimed at identifying preventable deaths and improving care, MPDSR requires a clear understanding of its components and how they function in practice [19, 21]. Sustainability, especially in low-resource settings, remains a challenge that needs further research [22–25]. Effective implementation also depends on local adaptation across all levels of the health system and is shaped by individual behaviors, organizational culture, and broader policy and political factors [26, 27].Most of the literature emphasizes tangible elements of implementation, such as tools and committee structures, and few studies have investigated individuals’ experiences, MPDSR dynamics and relational factors that underpin sustained practice [17, 23, 28].

To address the existing knowledge gap, we explored the components that enable the sustained practice of MPDSR, through the perspectives of stakeholders at national, subnational, and facility levels (Table 1).

**Table 1:** Country backgrounds of the MPDSR system

1. Nigeria: Nigeria offers insights from a populous Sub-Saharan African country grappling with significant maternal and perinatal health challenges. The adoption of the Maternal and Perinatal Death Surveillance and Response (MPDSR) system has been integrated into the broader quality-of-care (QoC) implementation framework, with ongoing efforts to strengthen its legal foundation MPDSR implementation in Nigeria began as part of a national response to the country’s high maternal mortality ratio, historically among the highest in the world. Prior to the launch of the national MPDSR programme, some states independently initiated maternal death reviews between 2008 and 2015. In 2013, the Federal Ministry of Health adopted maternal death review tools, protocols, and guidelines, enabling a national rollout with support from international partners [29, 30] By 2015, a National MPDSR Steering Committee and subcommittees were established, and the national implementation plan received approval from the National Council on Health. The first national MPDSR guidelines were also published that year [31]. In 2017 and 2018, individual states began releasing their first MPDSR annual reports Despite initial challenges such as inconsistent data collection and limited facility coverage, the MPDSR system in Nigeria has steadily expanded. The country’s decentralized, federal structure brings together a diverse array of stakeholders across national, state, and community levels. This complexity necessitates strong coordination to align MPDSR with ongoing federal health reforms, further supported by the proposed federal MPDSR Bill Nigeria’s approach emphasizes regular maternal and perinatal death reviews and community engagement to address preventable deaths. However, the long-term impact of these efforts remains insufficiently studied
2. North Macedonia: North Macedonia provides valuable insights from a transitioning healthcare system in Eastern Europe, particularly through the establishment of perinatal death reviews and a strong focus on perinatal care and quality improvement at the facility level. The country’s death audit system began to take shape following post-independence healthcare reforms in the 1990s In the early 2000 s, the Ministry of Health, with support from WHO and other European partners, initiated maternal death reviews in response to rising maternal mortality rates. The MPDSR system has since evolved, reaching key milestones between 2016 and 2019, when formal MPDSR structures were introduced. During this period, the focus was predominantly on perinatal death reviews, aimed at strengthening quality improvement efforts at the primary care level A major development occurred in 2019 with the establishment of the Perinatal Mortality Review Committee (PMRC) within the Ministry of Health. The PMRC was mandated to analyze all stillbirths and neonatal deaths occurring after 22 weeks of gestation and up to 28 days of life. In this study, we use the term “MPDSR” in reference to North Macedonia while acknowledging that its implementation is context-specific and focused primarily on perinatal death reviews North Macedonia’s emphasis on facility-level implementation reflects trends seen in other upper- and middle-income countries, where maternal and perinatal death surveillance tends to concentrate on hospital-based interventions [32]. This facility-based model aligns closely with quality improvement (QI) methodologies such as the Plan-Do-Study-Act (PDSA) cycle, which enables more immediate, localized responses to death reviews. However, it may lack the broader systemic impact that can result from national-level interventions [33]. This also illustrates ongoing debates in the quality of care field between assurance-oriented (measurement) and improvement-oriented (process) approaches [34]
3. Sri Lanka: Sri Lanka represents a country with a relatively advanced healthcare system and a comprehensive, systematized implementation of the MPDSR (Maternal and Perinatal Death Surveillance and Response) process. It is one of the earliest adopters of maternal death surveillance globally, with efforts beginning as early as the 1950s. Building on a long-standing commitment to maternal and child health, maternal death reviews were formally incorporated into national healthcare policy by the 1980s Sri Lanka’s notable success in reducing maternal mortality has been largely attributed to the effective use of MPDSR data to inform policy decisions and guide improvements in healthcare delivery [35]. In recent years, MPDSR monitoring efforts have expanded further. A web-based perinatal data collection system was launched in 2019 using the DHIS2 platform, and a national population-based stillbirth register has been operational since 2022 [36]. These innovations have positioned Sri Lanka as a model for other countries seeking to strengthen their maternal and perinatal death surveillance and response systems The structure of MPDSR implementation, across national, subnational, and facility levels, reflects broader differences in health system organization and governance when compared with other countries. Sri Lanka’s centralized health governance model enables strong national oversight by the Ministry of Health, which facilitates the enforcement of standardized MPDSR guidelines and promotes a culture of accountability. This centralized approach supports uniform adherence to maternal and perinatal health protocols and fosters systemic improvements Sri Lanka has consistently prioritized quality improvement through the continuous monitoring and review of maternal and perinatal deaths, using findings to refine health systems, strengthen service delivery, and optimize outcomes for women and newborns

## Methods

### Study design

This study employs a qualitative approach to understand MPDSR across diverse healthcare settings in Nigeria, North Macedonia and Sri Lanka. The methodological orientation used to underpin the study is content analysis [37].

The consolidated criteria for reporting qualitative research COREQ [38] were utilized to ensure transparent and rigorous reporting of our study (Supplementary file 1).

### Setting

Between 2022 and 2023, the World Health Organization (WHO), in collaboration with the global MPDSR Technical Working Group, conducted eight country case studies to document how MPDSR implementation has evolved across diverse contexts to improve maternal and perinatal survival and well-being [11]. This study focuses on three exemplar countries, Nigeria, North Macedonia, and Sri Lanka (Table 1), to explore the key components that have enabled sustained MPDSR implementation. We selected Nigeria, North Macedonia and Sri Lanka as exemplar countries on the basis of their sustained implementation over more than a decade, their integration of MPDSR into a broader quality improvement strategy, their geographical diversity and healthcare system characteristics (centralized versus decentralized), and quality of the data. Furthermore, these three countries differ in terms of mortality and are at different stages of the obstetric transition process [39], which makes them interesting cases to study. In Table 2, we report on their MMR, NMR and SBR levels and contexts.

**Table 2:** Indicators of mortality, population and essential maternal and newborn health services by country

Indicator	Nigeria	North Macedonia	Sri Lanka
MMR: number of maternal deaths/100,000live births (uncertainty interval (UI))^1^	993 (718–1540)	3 (1–5)	18 (15–25)
NMR: number of newborn deaths during the first 28 completed days of life per 1000 live births (UI)^2^	34 (23–51)	1 (1–2)	4 (3–5)
SBR: number of babies born with no signs of life at 28 weeks* or more of gestation, per 1,000 total births (UI) ^3^	23.9 (14.3–40.1)	3.6 (3.0–4.4)	5.9 (5–6.9)
Population ^4^	224 000 000	2 000 000	22 000 000
ANC 4 + : Antenatal care coverage at least 4 visits (%)	56.8% ^5^	95.7% ^7^	92.5% ^10^
SBA: proportion of births attended by skilled birth attendants (%)	50.7% ^6^	100% ^8^	99.5% ^11^
PNC maternal: Proportion of mothers who had postnatal care within 2 days of delivery (%)	41.8% ^5^	93.5% ^9^	99.2% ^11^
PNC newborn: Proportion of newborns who had postnatal care within 2 days of delivery (%)	% ^5^	98.6% ^9^	not reported ^11^

1. Trends in maternal mortality estimates 2000 to 2023: estimates by WHO, UNICEF, UNFPA, World Bank Group and UNDESA/Population Division. Geneva: World Health Organization; 2025. Licence: CC BY-NC-SA 3.0 IGO

2. Levels and trends in child mortality, report 2024: estimates developed by the United Nations Interagency Group for Child Mortality Estimation. New York: Unicef; 2025

3. Standing up for stillbirth: current estimates and key interventions. Report of the United Nations Interagency Group for Child Mortality Estimation, 2024. New York: Unicef; 2025

4. UN Population Division Data Portal [online database]. New York: United Nations; 2023 (https://population.un.org/dataportal/home, accessed 30 October 2023)

5. Nigeria Demographic and Health Survey 2018. Abuja, Rockville, Maryland, USA: National Population Commission (NPC) [Nigeria], ICF International; 2019

6. National Bureau of Statistics (NBS) and United Nations Children’s Fund (UNICEF). August 2022.Multiple Indicator Cluster Survey 2021, Survey Findings Report. Abuja, Nigeria: National Bureau of Statistics and United Nations Children’s Fund

7. State Statistical Office and Unicef. 2018–2019 North Macedonia Multiple Indicator Cluster Survey and 2018–2019 North Macedonia Roma Settlements Multiple Indicator Cluster Survey, Survey Findings Report. Skopje, North Macedonia: State Statistical Office and Unicef; 2020

8. Statistical Yearbook of Republic of North Macedonia, 2022. Skopje: State Statistical office (Republic of North Macedonia); 2022

9. North Macedonia 2018–2019 Multiple Indicator Cluster Survey

10. Department of Census and Statistics (DCS), Ministry of Healthcare and Nutrition (MOH). Sri Lanka Demographic and Health Survey 2006–07. Colombo, Sri Lanka: DCS, MOH; 2009

11. Sri Lanka Demographic and Health Survey 2016. Battaramulla: Department of Census and Statistics (DCS), Ministry of Health, Nutrition and Indigenous Medicine; 2017

### Study participants

In each country, 15–25 key informants were purposively selected on the basis of their roles and expertise in MPDSR implementation at different levels of healthcare. Table 3 presents the number of key informants by country, level and participant group. They were selected to cover the national, subnational and facility levels and based on their roles and involvement in MPDSR implementation in the country. The participants included participants such as policymakers, healthcare providers, public health officials, and international organizations involved in maternal and perinatal health in all three countries. All participants were invited to an individual interview to understand MPDSR implementation in their countries.

**Table 3:** Key informant interview details by country and participant codes

Countries	National level	Subnational or State level	Facility
Sri Lanka Total of 21 interviews	Ministry of Health national level (*5 interviews*) Professional association National (*4 interviews*) UN national personnel (*3 interviews*)	Subnational level (*5 interviews*)	Facility (*1 interview*) University hospital (*3 interviews*)
Nigeria Total of 25 interviews	Ministry of health national level (8 *interviews*) Professional association National (*2 interviews*) UN national personnel (3 interviews) Foundations and other organizations at national level (*4 interviews including one from university*)	State Level (*5 interviews*) UN subnational personnel (*1 interview*)	Facility (*1 interview*) University Hospital (*1 interview*)
North Macedonia Total of 15 interviews	Ministry of health national level (*4 interviews*) Professional association National (*1 interview*) Foundations and other organizations at national level (*1 interview*)	Subnational level (*1 interview*)	Facility (*8 interviews*)

### Data collection

A comprehensive research protocol was developed, outlining the study's objectives, methodology, ethical considerations, and data analysis procedures. The data collection tools included a semi-structured interview guide (supplementary File 2) based on the MPDSR cycle defined in the WHO guideline [8] and was developed by three authors, FP, MM, and AM. The key areas of inquiry were historical background, key participants, political commitment, implementation, healthcare providers, review and analysis, data management and follow-up actions, inclusion of perinatal deaths, funding and moving forward.

Data was collected from April 2022 to December 2023. After receiving the signed consent form of the participants, interviews were conducted in person (at the workplace) or through a digital meeting platform, with or without a video application, all with the exclusive presence of the interviewer and participant. Repeat interviews were not carried out. Each interview lasted between 45 and 60 min, and data saturation was discussed among the interviewers during data collection.

The interviews were conducted in English by SO and KJ and in Macedonian by GT and translated into English. All the interviews were audio-recorded and transcribed verbatim. Notes taken by interviewers during and after interviews were also checked to confirm the analysis. The transcripts were not returned to the participants.

### Data analysis and findings

Content analysis, following the approach outlined by Graneheim and Lundman [37], was applied for data analysis. The analysis commenced with coauthors FP, PM and AB identifying 'meaning units', whereby text was isolated from context and condensed into concise items while retaining the original meaning. FP and EM organized and listed the meaning units and transferred them to the Miro platform [40] to initiate collaborative analysis with the coauthors. FP, PM, AB and EM set up the analysis in Miro, screening all meaning units and further creating “condensed meaning units” which were answering the research question. The data analysis continued inductively with the identification of subcategories and categories, which were later organized into themes (Table 4). This process continued until no more categories emerged, after which the meaning units were re-evaluated and compared with others and with the interviewers for enhanced trustworthiness. This process ensured that the "enabling components" of the MPDSR system were identified inductively, grounded primarily in participants' narratives rather than guided by an external framework. During analysis, divergent or conflicting data were not dismissed as outliers; instead, they were coded separately and examined in depth. The research team actively sought disconfirming evidence to enhance analytical rigor, and these contrasting perspectives were integral to shaping interpretations of consensus, variation, and the overall thematic structure.

**Table 4:** Enabling components, categories and subcategories for the research question

Enabling components of MPDSR	Categories	Subcategories
1. Coordination of the MPDSR “programme” through committees by and across levels	Category 1.1: MPDSR is clearly defined by processes implemented by the committee at different levels with the use of guidelines and tools	Six steps of the MPDSR cycle
		National guidelines, policies and legislation
		Materials to support MPDSR
		Committee meetings, timeline and level
		Required members and levels participating and committees’ composition
	Category 1.2: Inclusion of perinatal component welcomed	Perinatal Reporting and Review
	Category 1.3: Coordination improves with a functioning supervision structure and budget	Reporting and coordination
		Internal supervision of the MoH or healthcare public system
		External technical support and collaboration needed
		Budget
2. Adoption and integration of a data management and analysis system	Category 2.1: Data collection and integration with existing data management systems	Data management
		Data analysis
	Category 2.2: Formulation of recommendations, monitoring and evaluation	Recommendations
		Data Monitoring, evaluation and follow up
	Category 2.3: Dissemination and publication of results become key "decision-making meetings"	Publication of results
		Dissemination
	Category 2.4: Capacity building for MPDSR committees covering data management and analysis, promoting a no-blame approach, and the implementation of recommendations	Training
3. A confidential, nonpunitive approach supported by committed leadership	Category 3.1: Confidentiality and nonpunitive approach foster a collaborative work environment	Blame culture
		Confidentiality
	Category 3.2: Positive leadership and champions are essential to enhance advocacy at all levels	Leadership
		Advocacy
4. A multilevel, country-specific response strategy integrating to broader health system strengthening	Category 4.1: MPDSR response implementation by level and beyond the local quality improvement	What is implementation of MPDSR response by level
		Integration and harmonization with quality-of-care initiatives

### Reporting

The analysis was organized by country and level (national, subnational and facility) to allow for context-specific descriptions and discussion. Participant quotations are presented to illustrate the findings, and each quotation is identified with a participant code, as shown in Table 3.

Major themes and categories are presented in the results, and consistency was ensured between the data and the findings presented.

### Research team and reflexivity

The interviewers (SO, GT and KJ) were all medical doctors with more than two decades of experience in public health programming, research and MPDSR. They were hired by the World Health Organization (WHO) as consultants to conduct interviews in each country: KJ in Sri Lanka, GT in North Macedonia and SO in Nigeria. At the beginning of each interview, the interviewer declared his or her reasons and interest in the research topic.

The analysis was conducted by FP, AB, and PM. FP brings a clinical and public health background, with specific expertise in maternal and perinatal health. At the time of the study, FP held the position of Technical Officer for MPDSR at WHO Headquarters. This role offered valuable insights into global policy and implementation frameworks, but also carried the potential to shape interpretations through an institutional lens, particularly with regard to normative expectations around MPDSR functionality and uptake.

AB and PM are senior researchers with extensive experience in qualitative health research in Sweden and international contexts, including low- and middle-income countries. Their academic distance from MPDSR implementation structures allowed for a more detached, critical perspective on the data. At the same time, their backgrounds in global health and qualitative methodology may have influenced how power dynamics, health system structures, and stakeholder narratives were interpreted and prioritized.

CH is a clinician with over 30 years of experience in maternal and newborn health in Africa, where she has conducted quantitative and qualitative studies on improving the quality of care in terms of maternal and newborn health.

The research team was aware of these positionalities and approached the analysis with reflexivity, engaging in regular discussions to surface assumptions, challenge interpretations, and triangulate perspectives. This collaborative, reflexive process aimed to enhance the trustworthiness and credibility of the findings, by balancing insider and outsider viewpoints and critically interrogating the influence of the researchers' institutional affiliations, epistemological orientations, and contextual experiences on the analytical process.

#### Ethics submission

Ethical approval was obtained both at the global level with the ERC WHO ethics clearance ERC.0003897006649 and within each selected country to ensure adherence to ethical standards in research, including informed consent procedures and confidentiality safeguards. North Macedonia 03–1937/1; Nigeria NHREC/01/01/2007–25/07/2022; Sri Lanka EC 22 060.

## Results

Among all the recruited participants (Table 3), only one respondent from Nigeria and two from Sri Lanka dropped out of the interview process. In Sri Lanka, the majority of participants were at the national level (with experience at the peripheral level), particularly the Ministry of Health, professional associations, and UN agencies, reflecting centralized health governance. Nigeria included respondents mainly at the national and state levels, including a few representatives from international organizations, indicating the engagement of diverse participants. In North Macedonia, there were more interviews with facility-level respondents and medical doctors, especially from university hospitals, with fewer from the national and subnational levels.

**Fig. 1:**
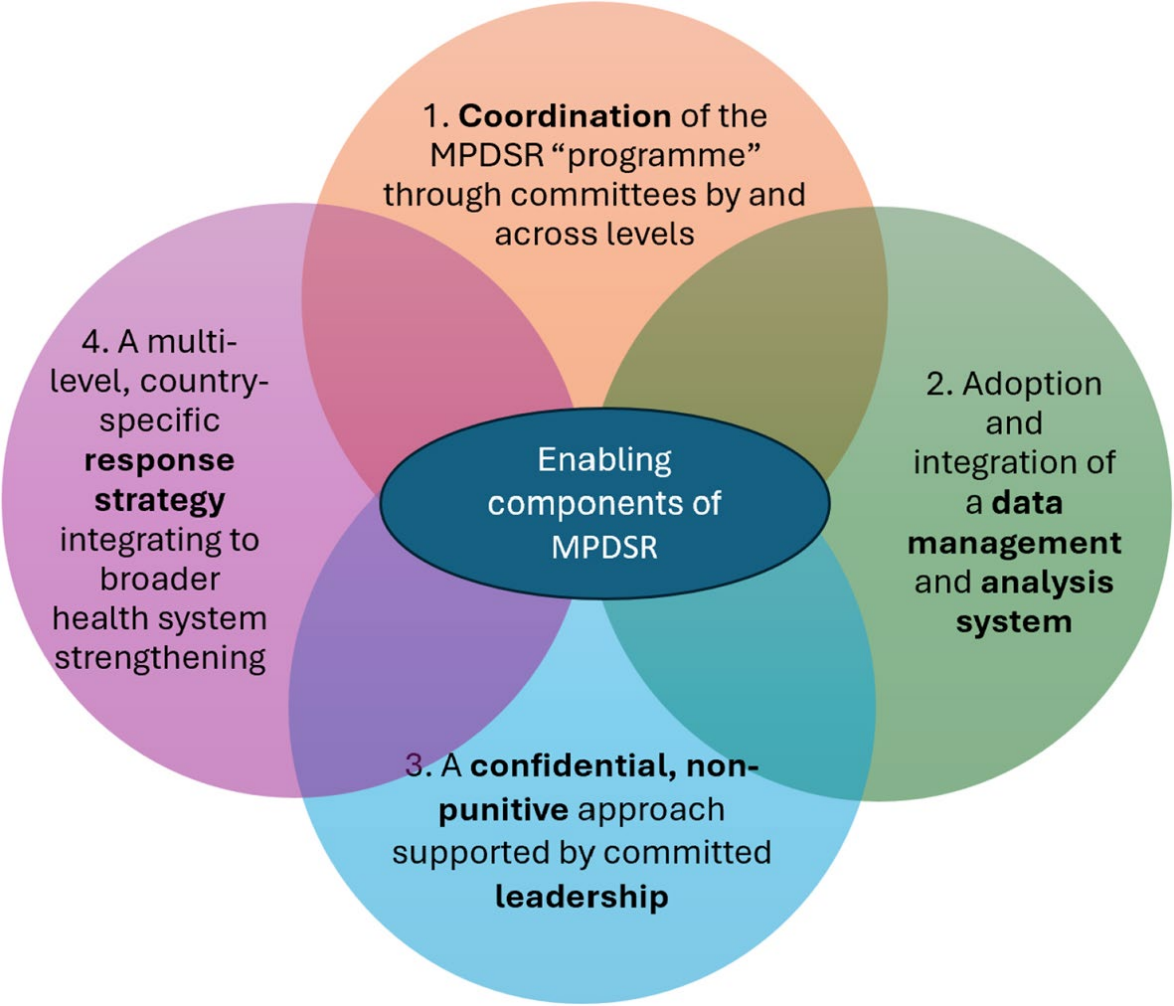
Enabling components of a sustained MPDSR implementation as understood by participants at the national, subnational, and facility levels

Participants at the national, subnational, and facility levels perceived the implementation of MPDSR as driven by four enabling components that form the foundation for the overarching theme “*MPDSR is sustained with increased accountability and responsibility by participants”.* These enabling components contribute to the sustainability of MPDSR, i.e., they make the MPDSR programme work over time, and all four enabling components are required for its implementation (Fig. 1). Detailed descriptions of each theme, or enabling components of MPDSR, are discussed in this section. Table 4 presents the enabling components with the linked categories and subcategories.

The MPDSR system is the engine that drives maternal and perinatal death reviews and follow-up. The enabling components (Fig. 1 and Table 4) are the fuel, structure, and road that allow the engine to run smoothly, stay on track, and reach its goals. Without the enabling components, the MPDSR system is likely to be fragmented, ineffective, or unsustainable. Without the MPDSR system, the enabling components lack purpose and direction. Together, they form a comprehensive ecosystem for improving maternal and newborn health outcomes (Table 5).

**Table 5:** Comparison between the MPDSR cycle and the MPDSR enabling components

Aspect	MPDSR system	Enabling components
Core function	Surveillance and response to deaths	Structural, cultural, and operational support
Focus	What the system does	What allows it to work effectively
Nature	Technical and procedural	Organizational, systemic, and value-based
Example of action	Case identification, case review, response	Forming committees, leadership support, building trust, system integration
Without it, what happens?	No mechanism to analyse and respond to deaths	System may exist in theory but fails in practice

Furthermore, we identified who is responsible to develop, maintain, and support the enabling elements for a sustained MPDSR system and the responsibility is shared across multiple actors (Table 6).

**Table 6:** Responsibilities of actors in support of the enabling factors of a sustained MPDSR implementation

Actor	Responsibility
Ministries of Health	Overall stewardship, policy setting, national coordination
Health facility managers and clinical leaders	Local coordination, data collection, and case review leadership
Development partners and technical agencies (e.g., WHO)	Capacity building, tool development, and strategic guidance
MPDSR committees at all levels	Operational oversight and response tracking
Professional bodies and civil society	Advocacy, accountability, and technical contributions

### Enabling component 1: coordination of the MPDSR “programme” through committees by and across levels

This component provides the structural and governance mechanism for MPDSR implementation. It ensures that MPDSR systems are not isolated within facilities but integrated across national, sub-national, and community levels, promoting accountability and consistency.

#### MPDSR is clearly defined by processes implemented by the committee at different levels with the use of guidelines and tools

Participants across three countries referred to MPDSR as a "program," with Sri Lanka and North Macedonia naming it the “safe motherhood programme.” Effective MPDSR implementation required structured guidelines, national policies, and coordinated committees across all levels. Establishing national guidelines was deemed essential for launching MPDSR. In Nigeria, a national committee emphasized cross-sectoral collaboration, whereas Sri Lanka and North Macedonia highlighted multilevel coordination through structured committees.

National and subnational participant inclusion was key for system-wide integration, with Sri Lanka and Nigeria being especially active. The participants from Sri Lanka and North Macedonia stressed the importance of clarity in MPDSR's six steps, national policies, guidelines, and committee roles. National guidelines aided implementation across all countries, with Nigeria debating MPDSR inclusion in the National Health Act for legal consistency. North Macedonia has a law supporting perinatal death reviews.

The participants held regular meetings to review MPDSR outcomes: Nigeria and Sri Lanka held quarterly and monthly national, state, and facility reviews, respectively; North Macedonia conducted national reviews every 1–3 months. Subnational reviews in Nigeria and Sri Lanka occurred quarterly, with Sri Lanka also reporting to higher levels after every 3–4 meetings and holding biannual district meetings and monthly facility reviews.

The participants described MPDSR as requiring multilevel engagement, including national, district, and facility participation. The inclusion of both national and subnational participants in national and facility committee meetings, especially in Sri Lanka and Nigeria, supports the integration of MPDSR into the wider health system. For example, in Sri Lanka, national-level activities involve a wide range of participants, including ministry officials and professional associations, and require national committees and collaboration with organizations such as UNICEF. At the district level, MPDSR involved regular meetings and investigations with district participation in national reviews. At the facility level, prompt death reviews with active participation from all involved health staff are needed. Community engagement included discussions on home-based deaths and required local government and community involvement.

Nigeria proposed a federal MPDSR Bill to mandate comprehensive maternal and perinatal death reviews with structured committees at the federal and state levels. The bill, which is waiting for approval, includes committee roles, confidentiality, legal protections, and reporting protocols. This legislation is seen as critical to improving MPDSR at the state level.

*For countries considering the enactment of similar legislation, it is crucial to engage all key stakeholders, including community representatives and beneficiaries. This inclusive approach ensures that decisions are based on informed opinions and avoids assumptions or misconceptions (United Nations subnational officer, Nigeria).*

#### Inclusion of perinatal components

In all countries, perinatal death reviews were introduced after maternal death reviews, with North Macedonia’s system focused primarily on perinatal deaths. The participants highlighted the importance of timely perinatal death reporting at the facility level within the MPDSR framework., Nigeria’s guidelines require reporting perinatal deaths within 24–48 h and reviews within a month. In Sri Lanka, perinatal death reviews require reports to be submitted nationally within 24 h. North Macedonia mandates 24-h perinatal death registration with legal requirements enforcing the implementation. However, the perinatal component remains at an early stage in Nigeria and Sri Lanka, as progress has not extended beyond the initial phase of notification and review.

While all included countries incorporate perinatal death reviews in MPDSR, only Sri Lanka reported integrating maternal and perinatal reviews under the same committee. This combined analysis provides insights into causes and modifiable factors, helping to prevent future deaths. As one participant remarked,

*"Perinatal and maternal death reviews need to be interlinked... Often, perinatal deaths are linked to issues found during ANC [antenatal care] or outside pregnancy. Proper investigations require stakeholders with broader knowledge." (Doctor of university hospital, Sri Lanka)*

In Nigeria and North Macedonia, respondents reported that only a sample of perinatal deaths was reviewed. In some Nigerian states, there are specific targets for perinatal death reviews and comprehensive child death audits are integrated into MPDSR.

North Macedonia mandated perinatal death reviews within 15 days, although only every fifth case was reviewed. A perinatal commission oversaw the data collection and review process at the facility level.

*"Maternal mortality cases are all reviewed, while perinatal cases are randomly reviewed, one out of every five." (Ministry of Health officer, North Macedonia)*

#### Coordination improves with a functioning supervision structure and budget

The participants in all three countries highlighted coordination and collaboration as essential to the success of MPDSR, with support from international organizations. The coordination and supervision structure are managed by the MoH in each country and has two aims: 1. to exchange data and information between the facility and subnational/national levels and 2. to organize the response at all levels. Data was shared from facilities to subnational and national levels, maintaining regular communication with the MoH, who further analyzed the data and provided feedback to facilities. A supervision structure also ensures regular national committee meetings (including subnational meetings in Nigeria), guiding the development of policy and guidelines as a result of escalating recommendations beyond hospital implementation.

Budget constraints are a common challenge. Participants in Nigeria and North Macedonia called for dedicated funding for MPDSR, whereas in Sri Lanka, they reported that MPDSR-related expenses were covered by the Ministry of Health budget.

### Enabling component 2: adoption and integration of a data management and analysis system

This refers to the backbone of evidence-based decision-making within MPDSR. Timely, accurate, and actionable data are essential for identifying causes of death, tracking trends, and evaluating the effectiveness of interventions.

#### Data collection and integration with existing data management systems

Participants across all countries highlighted key elements for MPDSR data collection and their integration with existing data management systems, including the use of correct classification of causes of death via the international classification of diseases (ICD) and the identification of underlying causes of death and modifiable factors; data validation of information collected during death case reviews; triangulation of data from multiple sources to address underreporting; integration of MPDSR data into existing health management information systems (HMISs) to avoid duplication; and electronic systems for data collection, improving accuracy and efficiency, with secure data management.

In Nigeria respondents recommended the synchronization of the MPDSR data flow and the routine health management information system. The MPDSR system used a separate data flow based on the Network of Obstetric Quality Assurance (NOQA) platform.

In North Macedonia, standardized data collection is managed by doctors and epidemiologists, where training and mentoring have been conducted to improve the correct classification of causes of death.

*In the MPDSR, there is better coding of the main causes of death. Previously, all the children were in one basket, and the main cause of death was prematurity. (District level officer, North Macedonia).*

In North Macedonia, facility-level respondents underscored the importance of distinguishing causes of death, such as prematurity, from other causes and determining factors. Detailed analyses revealed data gaps, such as missing information on head circumference, resuscitation methods, and infusion times. Efforts have focused on university clinics, which handle critical cases with higher mortality rates. Data analysis, led by the Institute of Maternal and Child Health, emphasized clinical improvements, although epidemiologists faced challenges in identifying perinatal death causes owing to physician misclassification, emphasizing clinicians' responsibility to ensure accurate reporting at the national level, while the statistical office oversees the database.

In Sri Lanka, MPDSR strengthened perinatal reporting across all levels including national-level confidential inquiries. Provincial and district data management are fed into the national maternal death database. Institutional protocols, training, and pathologist involvement in MPDSR reviews ensured data accuracy.

*… The Family Health Bureau issued a circular to regulate perinatal death review meetings, with the development of a format for pathological postmortem… thanks to MPDSR, a birth defects surveillance program was initiated (National professional association officer, Sri Lanka)*

#### Formulation of recommendations, monitoring and evaluation

Although sustained MPDSR implementation involves formulating recommendations, participants provided little detail on how recommendations were formulated, monitored, and evaluated. In Nigeria, MPDSR committees use the three-delay model. Sri Lanka's MPDSR emphasized the development of recommendations through expert discussions, which were then disseminated and implemented at the district and national levels. In North Macedonia, MPDSR focuses on developing and disseminating recommendations and creating action plans. Monitoring involves tracking the implementation of recommendations and revising them as needed.

#### Dissemination and publication of results become key "decision-making meetings"

Recommendations and findings were shared across the three countries through national annual or biannual reports, conferences, and meetings to enhance advocacy and collaboration. The participants stressed the importance of sharing MPDSR findings across national, subnational, facility and community levels for learning and improvement. This sharing of information increased accountability and promoted awareness among different participants. In Sri Lanka, for example, annual dissemination seminars organized by the MoH have become key "decision-making meetings" for allocating resources for change. MPDSR findings were included in annual reports by the Ministry of Health, Family Health Bureau and hospitals and disseminated through conferences, review meetings and numerous media modalities.

#### Capacity building for MPDSR committees covering data management and analysis, promoting a no-blame approach, and implementing recommendations

The participants discussed how training is crucial to equip healthcare professionals for MPDSR activities, not just related to data management and analysis. In Nigeria, capacity-building efforts included national training on MPDSR, aiming to train over 80% of medical workers in each state, with regular refreshers due to high staff turnover, suggesting a heavy investment in the programme. Sri Lanka focused on institutional training that fostered a no-blame culture in mortality reviews, with support from both the national and international levels. In North Macedonia, extensive training was usually provided by international organizations or professional associations on death classification, clinical dilemmas, and the no-blame approach to ensure effective data collection and implementation of recommendations.

### Enabling component 3: a confidential, non-punitive approach supported by committed leadership

This addresses the organizational culture necessary for open and honest case reviews. A blame-free environment encourages healthcare workers to participate in reviews candidly, leading to better identification of systemic failures rather than individual fault.

#### Confidentiality and nonpunitive approaches foster a collaborative work environment

All the participants agreed on the importance of a “no name, no blame” approach to encourage openness and maintain confidentiality. In all three countries, particularly at subnational levels, participants highlighted that leadership from the Ministry of Health and professional bodies helped shift from a punitive culture to a more open, nonpunitive culture. One participant noted:

*“The MDSR was initially considered a fault-finding exercise, but later, it became a good platform to present problems and prepare for reviews” (District level officer, Sri Lanka).*

Initially, the fear of punishment and incomplete information hindered participation. However, in Sri Lanka, the shift to a nonpunitive approach was driven by changes in the composition of death review meetings, leading to greater openness and learning. Expanded committee participation, including hospital staff and healthcare workers beyond management, fostered ownership and accountability.

In Nigeria, education and sensitization efforts helped reduce the blame culture, particularly in overcoming community-level cultural and traditional beliefs about death. One participant emphasized:

*“Audits should not be seen as punitive measures but as opportunities to enhance service quality. The goal is to improve outcomes in terms of maternal and perinatal mortality” (Doctor of health facility, North Macedonia).*

#### Positive leadership and champions are essential for enhancing advocacy at all levels

Leadership and champions played a key role in reducing blame during MPDSR implementation. Hospital directors, professional associations, and political figures helped reenergize the process, maintain regular meetings, and drive advocacy efforts. A participant shared:

*“The reason why MPDSR succeeded in Sri Lanka was due to the leadership maintained by the Ministry of Health” (Doctor of university hospital, Sri Lanka).*

In Nigeria, regular participation from MoH leadership reenergized MPDSR meetings. In North Macedonia, committed individuals on the MPDSR committee helped transmit enthusiasm and motivate staff.

### Enabling component 4: a multilevel, country-specific response strategy integrating to broader health system strengthening

This ensures that MPDSR findings lead to context-relevant action. It allows for tailored interventions at facility, district, and national levels, avoiding one-size-fits-all solutions and fostering local ownership.

#### MPDSR response implementation beyond local quality improvement

The participants noted that MPDSR aims to improve care quality at the facility, community, subnational, and national levels, indicating spillover effects beyond reducing maternal and perinatal mortality as the ultimate goal. The key reported benefits from the MPDSR responses were (1) increased government commitment to maternal and newborn health, (2) established consistent data collection for mortality monitoring, (3) greater involvement of professional associations in capacity building and (4) community engagement in the prevention of health risks and complications.

Sri Lankan participants highlighted the role of MPDSR in improving staff development, fostering a culture of sharing, and updating national guidelines, making it an integral part of system and policy improvements.

In addition, participants across the three countries at all levels reported the use of the MPDSR system beyond the local facility and community levels, including national and subnational levels (i.e., guideline and policy development, health information system development, etc.), allowing the MPDSR to become a sustained program in the broader health system.

Participants across all countries described MPDSR responses as multilevel, with strategies implemented at the national, subnational, facility, and community levels.

National responses, particularly in Nigeria and Sri Lanka, are shaped by field visits and death reviews, leading to internal circulars and actions to reduce mortality. In North Macedonia, national-level reviews guide responses, with a centralized, data-driven approach to care improvements.

Subnational or state-level reviews of maternal and perinatal deaths in Nigeria and Sri Lanka have led to targeted interventions aimed at improving care practices, whereas in North Macedonia, subnational responses are driven mainly by national-level findings. The participants emphasized the importance of tracking interventions and monitoring reductions in maternal and perinatal deaths to demonstrate the program's impact.

MPDSR at facility level stimulated rather direct quality improvement responses, while the system supported at regional and national level stimulated health system strengthening, including referral and training together with an improved policy framework.

*At the district hospital, actions on maternal deaths were immediate, with circulars on perinatal care, ANC and PNC on the basis of MPDSR findings (District level officer, Sri Lanka).*

In Nigeria, community-level responses often involved engagement with women’s groups, where women’s group coordinators (WGCs) play a crucial role in helping women in recognizing dangerous signs during pregnancy. They also supported the reporting of community deaths and facilitated verbal autopsies. North Macedonia's community responses were adapted to local needs (on the basis of national findings by the MoH) but were less community-driven than those of Nigeria.

#### MPDSR response integration with broader health system strengthening

The integration of MPDSR within quality of care (QoC) initiatives is still a grey area for most countries. However, it was mentioned by participants from Nigeria, who argued that their MPDSR structure is integrated into the broader QoC framework under the Ministry of Health, aligning planning, technical, and financial aspects. While the MPDSR and QoC teams share personnel, MPDSR focuses on investigating maternal and perinatal death cases, whereas QoC primarily addresses clinical aspects on the basis of evidence-based interventions at the facility level. State-level participants in Nigeria view MPDSR as addressing a broader range of issues related to maternal and perinatal causes of death, including aspects at the community level.

*“MPDSR integrates clinical aspects and overall service provision activities, whereas the QoC focuses primarily on clinical aspects,” (State level officer, Nigeria).*

## Discussion

This study examined the implementation of MPDSR in Sri Lanka, Nigeria, and North Macedonia, identifying both shared elements and unique practices across these diverse settings. We identified four enabling components of sustained MPDSR implementation that captured participants’ experiences and understanding of MPDSR at different levels, with the overarching theme “*MPDSR is sustained with increased accountability and responsibility by participants”.* Together, these components support the integration of MPDSR within the broader health system, driving improvements in the accountability and quality of maternal and perinatal care.

Kinney et al.’s scoping review (2021) [16] identifies key implementation factors influencing the effectiveness of Maternal and Perinatal Death Surveillance and Response (MPDSR) systems in low- and middle-income countries (LMICs). These factors are derived from empirical studies and span across health system functions, policy environments, stakeholder engagement, and operational processes. Our framework presented in the results of this paper outlines four enabling components of MPDSR implementation, operationalizing the insights from Kinney et al.'s findings. These components can be viewed as practical applications or domains of intervention derived from the broader themes identified in Kinney et al.’s review.

Our findings can be organized in three different lenses: a service delivery lens, which includes the tangible inputs needed for MPDSR implementation; a societal lens, which includes constructs that focus on social understanding and relationships; and a systems lens, which includes constructs that emphasize change dynamics, with adaptive learning to contexts in ways that are not always anticipated.

### Service delivery lens: inputs needed for implementation

MPDSR implementation across Sri Lanka, Nigeria, and North Macedonia revealed common needs at various levels of service delivery. Effective implementation requires clear national guidelines and coordination, which support participants at the national, subnational, and facility levels.

All three countries demonstrated the importance of national guidelines adopted from the WHO’s MPDSR guidance to ensure standardized processes for maternal and perinatal death reviews. The lack of international guidance for selecting perinatal cases, however, has led each country to develop unique approaches. This gap illustrates the need for more comprehensive, multilevel guidance to align national and subnational operational standards.

Our findings also support the conclusions from a recent publication that it is important if national legislation backs MPDSR implementation, emphasizing the need for institutionalizing MPDSR through formal legislation to ensure sustainability [41].

Additionally, data management and capacity building are critical service inputs for MPDSR. Skills and knowledge in MPDSR implementation are mainly reported in the literature related to data collection and use [16]. Proper classification, validation, and dissemination of mortality data enable meaningful analyses and inform actionable quality improvements. This aligns with findings from the WHO progress report on reducing maternal and newborn deaths and stillbirths [42], which stresses the need for robust data systems to ensure accurate mortality reviews and inform actionable quality improvements. Other studies have also analyzed cases in which MPDSR data are integrated into national routine health information systems. For example, the Kenya death review collection forms are linked to the District Health Information Software version 2 (DHIS2) database, which facilitates regular reporting, entry, aggregation, and examination of maternal death data. Despite this integration, mortality indicators are among the most challenging and often inaccurate metrics reported in routine information systems, and DHIS2 in its current form may not be suitable in every country for accurate recording of deaths [43, 44]. South Africa’s Perinatal Problem Identification Program (PPIP) initially faced similar challenges in death reporting, which later improved as ownership and participants’ buy-in increased [24].

Capacity-building efforts in Nigeria focused on large-scale training, while Sri Lanka implemented hands-on approaches. However, gaps remain due to limited documentation and a lack of standardized training materials. Continuous capacity building, tailored to the needs of implementers, is essential to sustain effective MPDSR processes. Strengthening data management and training infrastructure can help streamline MPDSR processes, thus reinforcing their role in health system quality improvement. Moreover, fostering peer learning through exchange visits and collaborative platforms can enhance knowledge sharing and promote best practices. Embedding capacity-building initiatives within broader health system strengthening strategies, such as digital health integration, performance monitoring, and supportive supervision, can further institutionalize MPDSR and improve its long-term sustainability.

### Societal lens: social understanding and relationships

The societal aspect of MPDSR implementation focuses on how interactions and relationships among participants influence the program’s success. Our findings demonstrate that moving towards a nonpunitive, confidential approach in MPDSR reviews is crucial for fostering trust and accountability. Establishing a code of conduct, nurturing team relationships, and promoting individual awareness of roles and responsibilities are crucial to ensure a culture of “No Name, No Blame, and No Shame”, suggesting that the three countries are investing in several, albeit not all, of the ten strategies to overcome the blame culture [45].

The ideal scenarios presented by national-level respondents do not always reflect realities at the health facility level. The literature from Nigeria [29, 30] indicates that while maternal death reviews can provide meaningful insights for preventing further maternal deaths, their effectiveness is often hampered by discussions surrounding external issues and accountability concerns, especially in mixed-level meetings involving “higher” and “lower” professional cadres. Addressing these challenges requires investment in team building and communication training, as well as fostering strong teamwork and commitment among health facility champions.

Structured committee reviews at all levels are a known implementation factor pivotal in maintaining momentum for MPDSR sustained implementation participants [46], emphasizing the necessity of regular feedback loops for the success of mortality review processes.

Leadership and committed champions are critical for reinforcing a positive MPDSR culture. The participants in Sri Lanka and Nigeria highlighted the importance of leadership in driving successful implementation, aligning with the global literature that points to leadership as a fundamental factor for effective MPDSR [16]. Additionally, strong leaders, or "champions," play a crucial role in sustaining MPDSR, and a recent study extends this understanding by identifying specific traits and motivations of these individuals [23]. While leadership is vital, the roles of committee members and strategies to motivate staff are not explicitly covered in international MPDSR guidance.

Effective leaders motivate staff and engage communities, establishing an environment where quality improvements become a shared goal [47]. Learning from high-performing facilities, where management actively promotes MPDSR, could help scale effective practices across similar settings.

A supporting environment, including institutional behavior and organizational culture, is undoubtedly an enabling factor for the successful implementation of MPDSR. Proactive institutions, which promote learning, play crucial roles in improving services and quality of care [48]. Hospitals that prioritize staff well-being recognize that errors are unintentional and that even the most skilled healthcare professionals may struggle to deliver high-quality care if their working conditions are inadequate.

### Systems lens: triggers for change and adaptive learning

The study confirms the role of MPDSR in health care quality improvement, expanding beyond its original focus on reducing maternal and perinatal mortality. This aligns with findings from prior studies [16, 49, 50] that describe MPDSR not only as a mortality review tool but also as a mechanism to enhance clinical processes, accountability, and governance structures.

The study demonstrates that sustained MPDSR implementation links quality improvement efforts across multiple levels—from facility-based death reviews to subnational oversight and national policy development. Individual motivation, role commitment, and support for MPDSR are deeply intertwined with a broader dedication to quality improvement [51]. This interconnected approach, spanning service delivery, supervision, management, and policy-making, is crucial for ongoing quality enhancement. The response in MPDSR has previously been detailed with three fundamental aspects: providing capacity-building activities to refresh health workers’ knowledge on evidence-based practices, updating national guidelines and supporting further research on specific conditions [52].

Successful MPDSR implementation requires a systems perspective, encompassing triggers for change and adaptive learning mechanisms. Since the WHO launch of MDSR in 2013, then of MPDSR in 2016, processes have shifted from maternal death reviews to maternal and perinatal death surveillance and response to go “beyond the numbers” [7] and even more recently “beyond the guidelines.” MPDSR has demonstrated itself as an intervention that is able to undertake adaptive learning [23, 25, 53], as participants move towards operational strategies that emphasize sustainable, practical approaches to ongoing improvements. This shift underscores the need for practical, sustainable approaches to keep the MPDSR system effective, address systemic requirements, and foster ongoing advancements in maternal and perinatal care.

This study explores perceptions of MPDSR in relation to other quality improvement activities. In Nigeria, MPDSR is integrated with national Quality of Care (QoC) efforts, addressing systemic improvements beyond immediate service delivery, supported by the MoH and Global QoC Network [54]. In contrast, North Macedonia’s integration remains at the facility level, whereas Sri Lanka focuses on national programs and policies. This variation highlights the need for flexible, context-specific strategies and structured policies to embed MPDSR within broader QoC initiatives.

### Sustained implementation and systemic integration

Sustainability in evidence-based interventions (EBIs), such as MPDSR, is crucial for lasting improvements in maternal and perinatal health outcomes [55]. Effective MPDSR implementation demands continuous evaluation of local adaptations, context-specific challenges, and factors such as relationships and leadership that foster enduring impact. Thus, to improve maternal and newborn health outcomes, a greater focus on how to operationalize the sustained implementation of interventions is necessary to address context-specific challenges that extend beyond health facilities [56]. A recent article offers insights into initiating, expanding, and reinforcing perinatal audits in South Africa [24]. Although the national perinatal audit program has a long history, there is no perfect MPDSR system, and its ongoing sustainability and current structure cannot be taken for granted. To effectively monitor the uptake and longevity of MPDSR, including perinatal audits, research approaches are needed that explore context, local adaptations, and important factors for sustainability, such as relationships, leadership, and trust.

Sustained MPDSR requires aligning efforts across the national, subnational, facility, and community levels to ensure long-term impact [16, 23]. Our study shows that key tasks such as case discussions, outcome monitoring, feedback, and funding allocation rely on coordinated action to reduce maternal and perinatal mortality. While international guidance often emphasizes facility-level work [8, 15], the three countries studied have scaled up MPDSR at the national level. Owing to the lack of standardized guidance, each country has developed its own approach with varied success. Clearly, multilevel guidance is essential for embedding MPDSR into quality improvement frameworks to enhance health outcomes and resource efficiency.

### Methodological considerations

The strengths of this study lie in its diverse case studies, which provide rich insights into different healthcare governance structures and enhance the generalizability of findings. The research offers a nuanced understanding of MPDSR's operationalization and sustainability challenges by including perspectives at the national, subnational, and facility levels. Additionally, it identifies essential components for sustained implementation, which can serve as a structured model for other countries.

This study has several limitations. We used three interviewers with similar backgrounds but different experiences in qualitative data collection methods because they needed to be familiar with the language and context. This background may have influenced how the interviewers probed and formulated the questions, which could have influenced the research findings. To reduce this influence, the data collectors were trained prior to the interviews and used all the same data collection tools. We did not undertake any result validation meetings with participants; however, we mitigated this by rigorous checks of information by interviewers, with their notes and transcripts. Due to the purposive sampling, the type of participants varied notably across countries, with Sri Lanka and Nigeria including a higher proportion of national-level stakeholders, while North Macedonia had a greater number of facility-level participants, which may have influenced the depth and focus of the data collected and contributed to the emergence and emphasis of certain themes within each country’s findings. Furthermore, participants at community level (although not widely implemented in all countries) were not included as study participants, which limits the collected information on community-level implementation. Finally, the list of the enabling components is not exhaustive, but it highlights foundational elements required for effective MPDSR. Other important aspects may include health workforce capacity, legal and regulatory environments, community engagement, and financial resourcing. However, these may be integrated within or support the four core components.

### Implications for policy and practice

Our study indicates that for a sustained implementation, enabling components are needed, which are currently not detailed in the present guidelines at all levels, such as the following: 1. Coordination of the MPDSR “programme” through committees by and across levels, which includes improved legislation, guidelines, and death review tools; 2. Investment in a strong data management and analysis system; 3. committed leadership to address the blame culture and ensure confidentiality; 4. A multilevel, country-specific response strategy integrated within the broader health system. Improved guidance to operationalize the integration of MPDSR within health systems and quality-of-care initiatives across all levels could help countries develop stronger MDPSR systems. Policymakers should consider developing clear multilevel operational strategies and plans to align MPDSR activities across national, subnational, and facility and community levels, enhancing data flow, coordination, and accountability. National and subnational health authorities should invest in developing context-specific training curricula, establish systems for regular mentorship and supervision, and ensure the availability of updated guidelines and tools.

### Priorities for future research

Among the enabling components of sustained MPDSR implementation, the third and fourth highlight crucial elements such as leadership, team trust, and the capacity to develop and execute response strategies, areas that remain underexplored in the existing literature. Future research should prioritize exploring team dynamics and relationships within MPDSR committees to uncover strategies for establishing psychological safety, trust and leadership and enhancing collaboration and improvements in diverse or resource-limited settings.

Additionally, developing a multilevel, country-specific implementation strategy requires further exploration, particularly in formulating recommendations and monitoring follow-up actions. Such frameworks could offer clear guidance to countries on operationalizing MPDSR, including more rigorous criteria for selecting perinatal death cases for review. Furthermore, assessing the potential integration of MPDSR with quality-of-care initiatives will clarify best practices and highlight areas for improvement. Finally, investigating sustainable funding and operational models is critical for understanding how MPDSR can be effectively sustained, especially in low- and middle-income countries reliant on international support.

## Conclusion

The implementation experience of MPDSR in Nigeria, North Macedonia, and Sri Lanka offers valuable insights for participants within these countries and for others aiming to expand and strengthen their own MPDSR programs. Participants’ understanding of MPDSR at different levels demonstrates the potential of MPDSR as a health system programme that goes beyond mortality reduction to improve health care quality and accountability at multiple levels. The sustained implementation of MPDSR requires a structured, multilevel approach that is interconnected with the broader health system. The enabling components of a sustained MPDSR implementation could help countries identify issues in their MPDSR system and support its implementation.

These findings echo the literature and provide more evidence to showcase these enabling components and their application for sustained MPDSR implementation. Particular attention is given to integration with quality improvement strategies, ensuring that MPDSR contributes to systemic quality of care improvements across contexts.

## Supplementary Information

Supplementary Material 1

## Data Availability

All data generated or analyzed during this study are included in this published article and its supplementary information files. Country case studies will be available in the WHO publication Maternal and perinatal death surveillance and response: global report on decade of implementation. Geneva: World Health Organization; 2025.

## Abbreviations

MPDSR: Maternal and Perinatal Death Surveillance and Response
QoC: Quality of care
QI: Quality improvements
PDSA: Plan‒do‒study‒act
WHO: World Health Organization
MoH: Ministry of Health
LMICs: Low- and middle-income countries
MMR: Maternal mortality ratio
NMR: Newborn mortality rates
SBR: Stillbirth rate
ANC: Antenatal care
SBA: Births attended by skilled health personnel
PNC: Postnatal maternal and newborn care
NOQA: Network of obstetric quality assurance
DHIS2: District Health Information Software version 2
PPIP: Perinatal Problem Identification Program
