# Supplementary material for: Beyond the guidelines: participants’ perspectives on sustained MPDSR implementation in Nigeria, North Macedonia, and Sri Lanka
Source: BMC Pregnancy Childbirth. 2025 Oct 6;25(Suppl 1):1015. doi: 10.1186/s12884-025-08180-0 (PMC12498454; doi:10.1186/s12884-025-08180-0)
Supplement: Supplementary file 1 — Supplementary Material 1 [file 12884_2025_8180_MOESM1_ESM.pdf]

COREQ (COnsolidated criteria for REporting Qualitative research) Checklist

A checklist of items that should be included in reports of qualitative research. You must report the page number in your manuscript where you consider each of the items listed in this checklist. If you have not included this information, either revise your manuscript accordingly before submitting or note N/A.

Topic	Item No.	Guide Questions/Description	Reported on Page No.
**Domain 1: Research team and reflexivity**			
Personal characteristics			
Interviewer/facilitator	1	Which author/s conducted the interview or focus group?	Research team
Credentials	2	What were the researcher's credentials? E.g. PhD, MD	Research team
Occupation	3	What was their occupation at the time of the study?	Research team
Gender	4	Was the researcher male or female?	Research team
Experience and training	5	What experience or training did the researcher have?	Research team
Relationship with participants			
Relationship established	6	Was a relationship established prior to study commencement?	Research team
Participant knowledge of the interviewer	7	What did the participants know about the researcher? e.g. personal goals, reasons for doing the research	Research team
Interviewer characteristics	8	What characteristics were reported about the inter viewer/facilitator? e.g. Bias, assumptions, reasons and interests in the research topic	Research team
**Domain 2: Study design**			
Theoretical framework			
Methodological orientation and Theory	9	What methodological orientation was stated to underpin the study? e.g. grounded theory, discourse analysis, ethnography, phenomenology, content analysis	Study design
Participant selection			
Sampling	10	How were participants selected? e.g. purposive, convenience, consecutive, snowball	Participant sel.
Method of approach	11	How were participants approached? e.g. face-to-face, telephone, mail, email	Participant sel.
Sample size	12	How many participants were in the study?	Participant sel
Non-participation	13	How many people refused to participate or dropped out? Reasons?	Participant sel
Setting			
Setting of data collection	14	Where was the data collected? e.g. home, clinic, workplace	Setting
Presence of non-participants	15	Was anyone else present besides the participants and researchers?	Setting
Description of sample	16	What are the important characteristics of the sample? e.g. demographic data, date	Setting
Data collection			
Interview guide	17	Were questions, prompts, guides provided by the authors? Was it pilot tested?	Data collectior
Repeat interviews	18	Were repeat inter views carried out? If yes, how many?	Data collectior
Audio/visual recording	19	Did the research use audio or visual recording to collect the data?	Data collectior
Field notes	20	Were field notes made during and/or after the inter view or focus group?	Data collection
Duration	21	What was the duration of the inter views or focus group?	Data collection
Data saturation	22	Was data saturation discussed?	Data collectior
Transcripts returned	23	Were transcripts returned to participants for comment and/or	Data collectior

Topic	Item No.	Guide Questions/Description	Reported on Page No.
		correction?	
**Domain 3: analysis and findings**			
Data analysis			
Number of data coders	24	How many data coders coded the data?	Data analysis
Description of the coding tree	25	Did authors provide a description of the coding tree?	Data analysis
Derivation of themes	26	Were themes identified in advance or derived from the data?	Data analysis
Software	27	What software, if applicable, was used to manage the data?	Data Analysis
Participant checking	28	Did participants provide feedback on the findings?	Data analysis
Reporting			
Quotations presented	29	Were participant quotations presented to illustrate the themes/findings? Was each quotation identified? e.g. participant number	Reporting
Data and findings consistent	30	Was there consistency between the data presented and the findings?	Reporting
Clarity of major themes	31	Were major themes clearly presented in the findings?	Reporting
Clarity of minor themes	32	Is there a description of diverse cases or discussion of minor themes?	Reporting

Developed from: Tong A, Sainsbury P, Craig J. Consolidated criteria for reporting qualitative research (COREQ): a 32-item checklist for interviews and focus groups. *International Journal for Quality in Health Care*. 2007. Volume 19, Number 6: pp. 349 – 357

Once you have completed this checklist, please save a copy and upload it as part of your submission. DO NOT include this checklist as part of the main manuscript document. It must be uploaded as a separate file.

Supplementary file 2:Maternal and Perinatal Death Surveillance and Response – Country case study: Interview topic guides for key informants

Section 1 is for key informants in all countries. Section 2 is country specific (to be developed further when focus of case study is finalised)

Section 1:

Background:

1. Please describe your roles and responsibilities in the provision of maternal and newborn health in your country
    a. How long have you been involved and at what level(s)? (Community, facility, sub-national, national)
2. Have you been involved in maternal and perinatal death reviews? If yes,
    a. For how long, in what capacity(ies), and at what level(s)?
3. When were maternal and/or perinatal death reviews first implemented in your country?
    a. What was the rationale for deciding to implement death reviews?
    b. If maternal and perinatal reviews were introduced at different times, what was the rationale for deciding to implement them at different times?
4. What opportunities drove the initiation of MDSR and MPDSR within your country?
5. What steps were followed prior to beginning the implementation of MDSR and MPDSR within your country?

Key Stakeholders:

6. Who were the key individuals and organisations who initially influenced the decision to implement MDSR/MPDSR/maternal death review/Stillbirth reviews/neonatal death review within your country?
7. How much enthusiasm was there for MDSR/MPDSR/death reviews/stillbirth review at the outset? How did it vary between stakeholders?
8. Were there any concerns expressed by stakeholders at the outset? If yes, what were these concerns?
9. After deciding to implement MDSR/MPDSR/death reviews/stillbirth review/PDR, who were the key actors/organisations initially involved in driving implementation forwards?
10. In what capacity?
11. Who are the key actors/organisations who are currently engaged in MDSR/MPDSR/death review/stillbirth review implementation?
12. In what capacity?
13. Has enthusiasm and commitment by key stakeholders changed over time?
14. What factors have influenced this change?
15. Does this vary between stakeholders?
16. Are there any concerns expressed by stakeholders now? If yes, what are these concerns?

Political Commitment:

17. At the outset, how much political will/commitment towards MDSR/MPDSR was there from the Ministry of Health /Department of Health?
    a. Was there any individual(s) who in your opinion drove the process in the country? If yes, please describe
18. Has political will/commitment towards MDSR/MPDSR changed over time?
    a. If yes, please describe the changes and the underlying changes
    b. What have been the biggest challenges in ensuring political commitment?
    c. How have these challenges been addressed?
19. Are there national policies and legal frameworks that refer to MDSR/MPDSR?
    a. If yes, please describe how these were developed
20. Are there subnational policies and legal frameworks that refer to MDSR/MPDSR?

Implementation:

21. In which specific place (facility, community, district, region, capital city, etc) was MDSR/MPDSR first implemented within your country
    a. Why was this location preferred?
22. How has MDSR/MPDSR implementation expanded over time?
    a. If implementation is still restricted to selected areas, what are the plans for further expansion and the timelines for the roll out?
    b. What factors have thus far influenced the roll-out?
    c. At what levels of healthcare facilities (e.g., health centres, district hospitals) is MDSR/MPDSR being implemented now?
    d. What is the level of implementation of MPDSR review of maternal and perinatal deaths at home or in the community? Can you provide details of implementation including challenges?
    e. Does MDSR/MPDSR implementation cover areas of the country with humanitarian needs? If yes, please provide details

    f. Was MDSR/MPDSR implementation impacted by the Covid 19 pandemic? If yes, please describe the impact.
23. Are non-government owned/operated healthcare facilities (faith-based, NGO, private for profit) involved in MDSR/MPDSR implementation?
    a. If yes, please describe their involvement
    b. If not, were any attempts been made to involve non-government owned health care facilities in MDSR/MPDSR implementation and what have been the responses to these attempts?
24. What attempts, if any, have been made to sustain or increase local, subnational, and national commitment to MDSR/MPDSR over time?
    a. Please describe these attempts
    b. How successful have these been?
25. What have been the biggest challenges, obstacles, or barriers that have been encountered during the MDSR/MPDSR implementation process?
    a. What was done to try to address these challenges, obstacles, or barriers?
26. What have been the most successful achievements of MDSR/MPDSR implementation?
    a. What are you - as an individual - most proud of your country's achievements in MDSR/MPDSR implementation?
    b. Are there any achievements that could be replicated in other countries? If yes, please describe them.
27. What is the level of engagement of health professional associations in the MDSR/MPDSR processes? Do you feel that they are fully engaged and committed to the process? Can you give some concrete examples in the implementation? Do health professional association guidelines include MDSR/MPDSR as one of their priorities?
    a. Have MDSR/MPDSR processes been integrated in preservice training curricula? Or are they limited to in service training only?

Healthcare Providers and MDSR/MPDSR

28. Have healthcare providers been trained in MDSR/MPDSR?
    a. If yes, how many have been trained?
    b. When was the training provided and by whom?
    c. What cadres of healthcare providers have been trained in MDSR/MPDSR?
    d. Are healthcare providers at all levels of the health system been trained? If not, which levels have not been included?
    e. Have healthcare providers had refresher training in MDSR/MPDSR?
    f. What have been the biggest obstacles in providing MDSR/MPDSR related training?
29. What is/was the general attitude of health care providers towards MDSR/MPDSR
    a. Prior to training?
    b. After training?
    c. After implementation of MDSR/MPDSR?
    d. How confident are health care providers in implementing MDSR/MPDSR?
30. What roles do healthcare providers play in the various steps of the MDSR/MPDSR process?
    a. Do these differ by the level of healthcare facility? Please provide examples
31. Are individual healthcare providers worried about being blamed for maternal or perinatal deaths?
    a. If yes, could you please provide a typical example without identifying the individual healthcare provider, the healthcare facility, or the individual whose death was being reviewed?
    b. Has any action been taken to prevent individual health workers and facilities from blame and punitive actions following maternal and perinatal deaths?
    c. How successful have these activities been in addressing the "blame" culture?

Reviews & Analyses:

32. Are maternal/perinatal death reviews routinely conducted for <u>all</u> relevant deaths?
    a. If not, what are the main reasons for not doing so?
33. Within what time frame are maternal and perinatal deaths expected to be reviewed?
    a. What proportion of the reviews are consistently conducted within the correct time limits?
    b. What are the main reasons for not doing so?
34. Who participates in the death reviews at the different levels?
    a. Please describe the composition of the groups that participate in death reviews
    b. If you have participated in death reviews, please describe your experience of the process, and share any challenges that you faced
35. In what proportion of death reviews are the reviewers able to satisfactorily determine the causes of death and factors leading to the death?
    a. What are the main reasons for inability to determine causes and factors leading to death?
36. What proportion of recommendations made following death reviews are for
    a. Improving health seeking behaviours of individuals and communities?
    b. Improving transport and referral processes?
    c. Improving quality of care in facilities?
    d. Improving follow up care after discharge from facilities?

37. What proportion of recommendations made
    a. Are potentially achievable within 1-2 years? Please give an example.
    b. Are potentially achievable with existing resources? Please give an example

Data Management and Follow up Actions:

38. What system is used for recording MDSR/MPDSR data?
    a. How soon after death are data entered in the system?
    b. Who is responsible for data collection for deaths (a) in facilities (b) at home?
    c. Describe the methods used for data collection (a) in facilities (b) at home
    d. Who is responsible for data entry?
    e. How are data analysed? How often?
    f. Who is responsible for data analysis?
    g. Who maintains the database?
    h. How is confidentiality of data ensured?
    i. What data quality assurance mechanisms are in place?
39. Do MDSR/MPDSR data feed into any routine data systems? If so, please describe
    a. Are the national Health Management Information Systems (e.g. DHIS2), and the MPDSR database integrated? Or are they managed separately?
40. At what level are the completed reviews aggregated for analyses? (e.g., national, sub-national, facility)
    a. How often are the aggregated reviews analysed?
41. Is any information from MDSR/MPDSR data analyses fed back to facilities and communities?
    a. If so, what is process followed?
    b. How frequently is the information fed back?
    c. In general, what has been the response from facilities.
42. What are the key challenges to timely completion of:
    a. Reviews?
    b. Data analysis?
    c. Feedback?
43. What have been the key findings from the review process in the last two years?
44. Are there any networks, initiatives, or forums that MDSR/MPDSR findings feed into at the national level or subnational level?
    a. If so, please describe them and how they work with MDSR/MPDSR findings?
45. How are the findings of MDSR/MPDSR disseminated?
    a. If reports are published, how often and is what format are they available?
    b. Any dissemination meetings at national and subnational levels?
    c. Is the information shared through the media?
    d. Is the information shared through community platforms?
    e. What has been the general response in the country to the information?
46. What key actions have been taken to date in response to findings from the review process?
    a. Please give some examples describing the actions, who initiated it and when, and what were the outcomes
47. What are the key challenges and barriers to identifying and implementing remedial actions recommended after death review?
    a. Please give some examples
48. How is implementation of recommendations from the MDSR/MPDSR process monitored?
    a. What have been the key challenges in monitoring implementation? Please give some examples.
49. Are any of the MDSR/MPDSR data integrated within the national HMIS indicators? Are there MDSR/MPDSR related indicators that are included within the national HMIS? Is there a parallel digital data system in place to document MDSR/ MPDSR ?
50. Is there any data triangulation done to compare the results of the MDSR/MPDSR process with other maternal and perinatal death sources?

Inclusion of Perinatal Deaths (stillbirths and neonatal deaths)

51. Are perinatal deaths included in the review process?
    a. If yes, please clarify how perinatal deaths are defined.
    b. When did this begin?
    c. What process was followed for the inclusion of perinatal deaths?
52. How are perinatal deaths notified to the authorities?
    a. How soon after perinatal deaths and to what level should be information be sent?
    b. Please describe the process and any challenges with notification.
53. What process is followed in reviewing perinatal deaths?
    a. Are all perinatal deaths reviewed? If not, how are deaths selected for review?
    b. Does the review process differ from the maternal death review process? If so, how?

54. What challenges, obstacles, or barriers have been encountered while trying to incorporate perinatal deaths into the maternal death review process? Please provide examples
    a. What has been done to address these challenges, obstacles, or barriers?
55. If perinatal deaths are not being reviewed, are there any plans in place to include perinatal deaths with maternal death reviews?
    a. How important do key stakeholders think it is to incorporate perinatal deaths with the maternal death review process?
    b. How do their views vary?

Funding

56. What are the major sources of funding for MDSR/MPDSR implementation in your country?
57. Have funding restrictions hampered MDSR/MPDSR implementation plans?
    a. If so, how? Please give examples
58. Have there been any challenges with trying to obtain funding for MDSR/MPDSR?
    a. If so, describe these challenges
    b. Describe any strategies that have been used successfully to mobilise resources for MDSR/MPDSR?

Moving Forwards

59. In your opinion, what should be the priority activities in the future for MDSR/MPDSR implementation?
    a. Probe for extending implementation to areas not currently covered e.g., humanitarian settings, improving response, etc (depending on responses to earlier topics)
60. Does the current situation in our country facilitate initiation of these priority activities?
    a. What would be the facilitators for the priority activities?
61. What challenges do you anticipate in the present situation with conducting these priority activities?
    a. How do you propose to address those challenges?

Section 2 *Draft to be finalised after country study focus is finalised*

The Ministry of Health has proposed that the case study in this country focuses on ____________________________(Insert theme).

62. Are you familiar with this aspect of the work?
    a. If yes, please describe how you became familiar – how long, in what capacity
    b. Please describe the important aspects of this work that should be showcased.
        i. When did it start, who initiated it
        ii. Please describe any innovative approaches in this work
    c. Are you aware of the implementation costs for this work?
        i. If yes, please describe budget, and source of funding
    d. Were there challenges in implementing this specific work?
        i. What were the challenges? How were they addressed?
        ii. Who/what were the key enablers in implementing this work?
        iii. Who/what were the major barriers?
    e. Is this work continuing? If not, why did it stop?
    f. If the work is sustainable, what factors have contributed to its sustainability.
    g. What are the key lessons from this work that you would like to highlight?
    h. If other countries are keen to introduce similar work, what in your opinion are the key implementation issues those other countries should be aware of
    i. Are you aware of any published information on the work to be showcased? If yes, please describe
    j. Are you aware of unpublished data on this work that could be made available for this study – please provide details

63. If you are not familiar with the proposed topic, please describe other areas of work that you feel should be showcased.
    a. Describe why you think the topic you have suggested should be showcased
